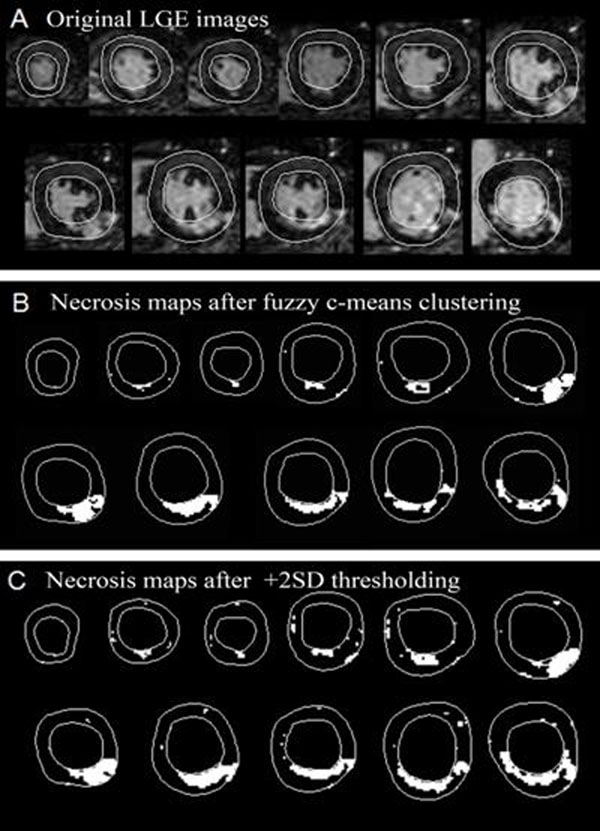# Fuzzy-logic, manual and semi-automated 2SD-based approaches for quantification of myocardial necrosis from late contrast enhancement magnetic resonance images: comparison with biochemical assessment of infarct size and left ventricular volumes and function early after myocardial infarction

**DOI:** 10.1186/1532-429X-13-S1-O106

**Published:** 2011-02-02

**Authors:** Nicolas Baron

**Affiliations:** 1 Centre Hospitalier de Versailles, Le Chesnay, France

## Purpose

Cardiac Magnetic Resonance Imaging (CMR) provides reliable estimation of the extent of necrosis after acute myocardial infarction (AMI). Nevertheless usual quantification methods are time consuming and subjective. We sought to assess necrosis extent with a new method (fuzzy c-means clustering), compared to usual methods, applied on Late Gadolinium Enhancement (LGE) sequences. Results were compared to biochemical markers of necrosis and LV functional parameters.

## Methods

CMR was performed 50±21 hours after AMI in 52 consecutive patients. We quantified the amount of necrosis using three approaches: 1) automated fuzzy c-means clustering method, 2) +2SD thresholding approach with manually defined remote myocardium, 3) +2SD thresholding approach with automated definition of the remote myocardium. Results were compared with biochemical marker-estimated necrosis amount and left ventricular (LV) functional parameters.

## Results

Although the 3 methods strongly correlated (r 0.89 to 0.99, p<0.0001 for all), the quantified amount of necrosis was significantly different between methods. Manual quantification reported highest (20.5±17.6%), whereas fuzzy c-means clustering reported lowest (12.5±12.1 %) values. Strongest correlations were found between fuzzy c-means and both biochemical quantification of necrosis (r 0.61 to 0.80) and LV functional parameters (r 0.55 to 0.71).

## Conclusions

The fuzzy c-means-assessed amount of necrosis on CMR images shows higher correlation with biochemical infarct size quantification as well as left ventricular global and regional function than usual methods. This method may be clinically useful in the evaluation of patients with AMI.

**Figure 1:**